# Human Breast Milk Exosomes: Affecting Factors, Their Possible Health Outcomes, and Future Directions in Dietetics

**DOI:** 10.3390/nu16203519

**Published:** 2024-10-17

**Authors:** Elif Çelik, Özge Cemali, Teslime Özge Şahin, Gülsüm Deveci, Nihan Çakır Biçer, İbrahim Murat Hirfanoğlu, Duygu Ağagündüz, Ferenc Budán

**Affiliations:** 1 Department of Nutrition and Dietetics, Faculty of Health Sciences, Süleyman Demirel University, Isparta 32260, Türkiye; elifcelik@sdu.edu.tr; 2 Department of Nutrition and Dietetics, Faculty of Health Sciences, Trakya University, Edirne 22030, Türkiye; dytozgecemali@gmail.com; 3 Department of Nutrition and Dietetics, Faculty of Health Sciences, Afyonkarahisar Health Sciences University, Afyonkarahisar 03030, Türkiye; tozgeyrsn@gmail.com; 4 Department of Nutrition and Dietetics, Faculty of Health Sciences, Çankırı Karatekin University, Çankırı 18100, Türkiye; gulsumdeveci7@gmail.com; 5 Department of Nutrition and Dietetics, Faculty of Health Sciences, Acıbadem Mehmet Ali Aydınlar University, Istanbul 34752, Türkiye; nihancakir@gmail.com; 6 Department of Neonatology, Faculty of Medicine, Gazi University, Ankara 06500, Türkiye; imh@gazi.edu.tr; 7 Department of Nutrition and Dietetics, Faculty of Health Sciences, Gazi University, Ankara 06490, Türkiye; 8 Institute of Physiology, Medical School, University of Pécs, H-7624 Pécs, Hungary

**Keywords:** human breast milk exosomes, breastfeeding, infant nutrition, nutrigenomics

## Abstract

**Background:** Human breast milk is a complex biological fluid containing multifaceted biological compounds that boost immune and metabolic system development that support the short- and long-term health of newborns. Recent literature suggests that human breast milk is a substantial source of nutrients, bioactive molecules, and exosomes. **Objectives:** This review examines the factors influencing exosomes noted in human milk and the impacts of exosomes on infant health. Furthermore, it discusses potential future prospects for exosome research in dietetics. **Methods:** Through a narrative review of the existing literature, we focused on exosomes in breast milk, exosome components and their potential impact on exosome health. **Results:** Exosomes are single-membrane extracellular vesicles of endosomal origin, with an approximate radius of 20–200 nm. They are natural messengers that cells secrete to transport a wide range of diverse cargoes, including deoxyribonucleic acid, ribonucleic acid, proteins, and lipids between various cells. Some studies have reported that the components noted in exosomes in human breast milk could be transferred to the infant and cause epigenetic changes. Thus, it can affect gene expression and cellular event regulation in several tissues. **Conclusions:** In this manner, exosomes are associated with several pathways, including the immune system, oxidative stress, and cell cycle, and they can affect the short- and long-term health of infants. However, there is still much to learn about the functions, effectiveness, and certain impacts on the health of human breast milk exosomes.

## 1. Introduction

Human breast milk is an ideal, irreplaceable nutrient for ensuring and maintaining infant health and development [1,2]. The World Health Organization [3], American Academy of Pediatrics [4], and European Society of Gastroenterology, Hepatology, and Pediatric Nutrition [5] have recommended exclusive breastfeeding for the first 6 months of life, followed by complementary foods until 2 years of age or older. The unique composition of human breast milk and its short- and long-term effects on health have been the subject of scientific interest and extensive research [6]. Despite all these studies on human breast milk, its uniqueness remains, as it contains components that are still being discovered. Although several commercial formula companies are attempting to produce formulas with a composition that substitutes human breast milk, developing a product that will completely replace human breast milk remains impossible owing to newly discovered ingredients and the difficulty in artificially producing the identified ingredients [7,8].

Human breast milk is “not just food”, it is a complex biological fluid containing multifaceted biological compounds, including carrier systems that promote immune and metabolic system development, supporting the short- and long-term health of newborns [9,10,11,12]. Human breast milk is essential for the intestinal maturation regulation and immune cell development [11,13]. During the postnatal period, adaptive immunity remains developing, while the intestinal epithelial barrier of the infant’s gastrointestinal tract matures. Immunoglobulins, leukocytes, stem cells, lysozymes, lactoferrins, and lactadherin are among the molecules detected in human breast milk that offer passive immunity for the infant and influence immune system development [11]. Moreover, these components positively support the microbiota and protect against pathogenic microbial infections [10,14].

In addition to macro- and micronutrients that have an impact on maternal and infant health, breast milk also contains many bioactive components such as extracellular vesicles [15]. Extracellular vesicles in milk, and especially their subgroup exosomes, contain lipids, proteins, mRNAs, long non-coding RNAs (lncRNAs), circular RNAs (circRNAs), and micro-ribonucleic acids (miRNAs) [16]. Exosomes are detected in most body fluids, including blood, saliva, urine, cerebrospinal fluid, lymphatic fluid, amniotic fluid, and human breast milk [17,18]. Human-milk-derived exosomes were first isolated in 2007 [19]. Subsequently, milk exosomes were isolated and characterized from cows, sheep, buffaloes, camels, and pigs [15,20]. Both milk and crop-milk contain exosomes that withstand digestion, and miRNAs enter intestinal cells (ICs), pass through the bloodstream, and reach the cells of other tissues. This bioactive compound reaches the nucleus [15,20].

Exosomes mainly function in transporting cellular components, including proteins, lipids, and nucleic acids, to recipient cells. They can influence multiple biological process-es within the recipient cells, including immunological function, immunological response, intercellular signaling, inflammation, stress defense stem cell growth and differentiation, neuronal function, cell signaling, tissue regeneration, and viral replication, which in turn affect human physiology and pathology [17,21,22,23]. Among the extracellular vesicles in milk, exosomes have attracted recent attention because the cargo in exosomes is a regulated structure and is a nonrandom process. Thus, exosomes play an essential role in cell-to-cell communication [24]. At the same time, RNAs in exosomes can affect epigenetic alterations [11,25]. This process involves heritable variations in gene expression that are controlled by histone modifications, the activation or silencing of genes linked to ncRNAs, and deoxyribonucleic acid (DNA) methylation, among other epigenetic modifications [25,26].

miRNAs are one of the main biological elements of exosomes [16,27]. Milk-derived miRNAs may also play possible functional roles in epigenetic regulation, intestinal health, immune regulation, and metabolic diseases [16]. However, there is still much to discover about the proteome of human breast milk exosomes [28].

This review examines the factors influencing exosomes noted in human breast milk and the impacts of exosomes on an infant’s health. Moreover, this review discusses potential future prospects for exosome research in dietetics.

## 2. Exosomes Derived from Human Breast Milk

Breastfeeding has advantages for the infant and the mother health, and the “Developmental Origins of Health and Disease” hypothesis suggests that environmental expo-sures during infancy can permanently affect health and disease risks later in life [11,29,30,31]. Breastfeeding offers short-term health benefits by preventing various diseases such as microbial infections, gastroenteritis, otitis media, sudden infant death syndrome, and other childhood illnesses [32].

Human breast milk is crucial for infant growth and development, containing 87–88% water, 7% carbohydrates, 1% protein, and 3.8% fat. It contains vitamins, minerals, bioactive factors, immune cells, stem cells, and more. Extracellular vesicles, exosomes, and miRNAs derived from breast milk are of increasing interest for transmission and development [15].

Exosomes have been identified in the structure of biological fluids, such as the saliva and plasma [33]. Moreover, exosomes are detected in human breast milk, which is essential for babies for years [34]. Exosomes, noted in several structures, are produced because of the pullulation of the plasma membrane, and play significant roles in intercellular signaling [35]. Providing communication between cells will ensure homeostasis regulation [36]. Therefore, understanding the role of exosomes in biogenesis and/or intercellular communication is imperative.

**Figure 1:**
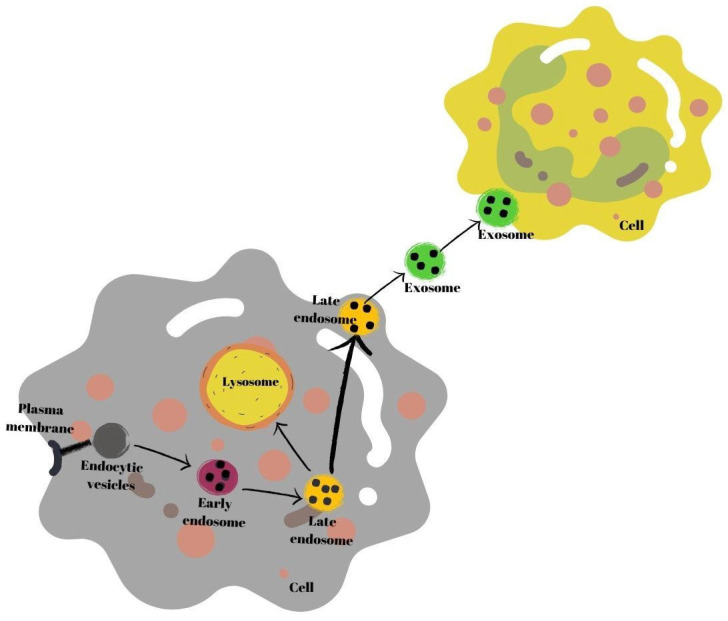
Schematic summary of exosome biogenesis.

Exosomes are secretory products of endosomal origin [35]. Early endosomes are produced when endocytic vesicles unite with the plasma membrane (multivesicles). Subsequently, the process of formation of late endosomes from early endosomes begins. In this process, endosomes in the endosomal membrane fold inward, and intraluminal vesicle structures are formed. Next, late endosomes either connect with the plasma membrane or with a lysosome, and break down and are secreted into the extracellular space as exosomes [37]. Secreted exosomes are absorbed by recipient cells. Exosomes may fuse with the plasma membrane of recipient cells or be endogenized by cells [38] (Figure 1).

Mammary gland epithelial cells secrete milk exosomes which are released by milk fat globules during lactation [7]. The characteristics make these exosomes crucial signaling molecules (signalosomes) between mothers and infants as they are one of the primary channels of communication between them [25,39].

### 2.1. Components of Human Breast Milk Exosomes

Exosomes, both autocrine and paracrine, play a fundamental role in the delivery of functional messages to cells [40]. Membrane vesicles with receptors to assure traffic specificity are used by cells to transport proteins, messenger RNAs (mRNAs), miRNAs, and other bioactive cargo molecules between intracellular organelles [7,28]. Exosomes are rich in mRNA, miRNA, DNA, lipids, and proteins [21,28,40,41,42]. Indeed, long-chain fatty acids, complex oligosaccharides, and bioactive proteins are the main components of human breast milk, and only several other bioactive substances were detected in human breastmilk [20]. The cargo in milk exosomes is regulated; thus, it is a nonrandom process [43]. The most noticeable differences between preterm and full-term milk regarding cytokines, lactoferrins, and growth factors are noted in preterm and colostrum milk. These differences typically last for 4 weeks following delivery. Exosomes are secreted as a package containing several components. Noncoding ribonucleic acid types (lncRNAs and circRNAs) and proteins (CD81) are noted in the package, that is, in the exosomes [35,36,44,45]. Moreover, miRNAs take an essential place. In human breast milk, more than 1400 different miRNAs have been identified [43,46,47].

Additionally, exosomes have properties specific to the cell, wherein they are secreted and contain surface proteins, membrane proteins, other proteins, DNAs, and RNAs [28]. A review by Chutipongtanate et al. [21] showed that bioactive peptides such as tetraspanins, lactadherin (milk fat globule EFD factor 8 (MFGE8)), transforming growth factor-beta (TGF-β), integrins, intracellular adhesion molecule-1, mucin-1, or proteins are in human breast milk exosomes [21]. An article written by Geddes and Kakulas [48] stated that human breast milk during lactation contains proteins (casein, whey proteins, cytokines, amylase, and growth factors), fats (short-, medium-, and long-chain unsaturated fatty acids), peptides (ghrelin and leptin), and carbohydrates (lactose and oligosaccharides) [48].

#### 2.1.1. Tetraspanins and Other Bioactive Proteins and Peptides

Tetraspanins (CD83, CD9, CD81, and CD63) are among the essential components of human breast milk [11]. Furthermore, tetraspanins, one of the transmembrane proteins, are noted in human breast milk exosomes. A study by Giovanazzi et al. [49] revealed the presence of CD3, CD14, CD9, CD24, CD29, CD44, CD63, CD105, CD133-1, CD146, CD326, and CD81 in human breast milk exosomes. Of these, CD326, CD14, CD133-1, CD24, CD146, and CD3 are the unique and essential tetraspanin types of human breast milk [33,40,49,50,51,52].

Furthermore, studies examining exosomal tetraspanins in different periods of human breast milk have been conducted [53,54]. A study by Liao et al. [53] revealed that CD9 was detected during the early, middle, and late lactation periods [53]. Wang et al. [54] performed a bioinformatic analysis of the differential expression of 70 peptides between CD9/CD63, preterm, and term extracellular vesicles, and the results showed that the peptides in pre-term milk undergo significant changes compared with those in term milk. Over time, 47 of them were upregulated, whereas 23 of them were downregulated [54].

#### 2.1.2. TGF-β

Another component of human breast milk exosomes is TGF-β [11]. A review con-ducted by Chutipongtanate et al. [21] reported that TGF-β also has an important place in exosome structure and plays a role in CD4^+^CD25^+^FoxP3^+^Treg differentiation [21].

TGF-β levels may vary depending on various lactation periods. Exosomal TGF-β2 levels, one of the TGF-β types, may vary depending on breastfeeding stages. A study by Qin et al. [55] reported that although there are expressions of TGFβ1, TGFβ2, matrix met-alloproteinase (MMP) 2, MMP3, and MMP9 in human breast milk exosomes, these are higher during the early human breast milk period than during the whole human breast milk period [55].

#### 2.1.3. Noncoding RNAs

Human breast milk exosomes likely carry genetic materials, including noncoding RNAs, and they participate in cell-to-cell communication since they are known to control significant gene pathways. A study by Lässer et al. [33] suggested that human breast milk exosomes can transmit functional genetic but mostly epigenetic signals to other cells since exosomes can transfer their RNAs to target cells [33].

Although miRNAs constitute approximately 15% of noncoding RNAs in human breast milk exosomes, the remaining percentage of noncoding RNAs are lncRNAs, circRNAs, ribosomal RNAs, and transfer RNAs [32]. A study by Rubio et al. [56] revealed that various types of small RNAs, including miRNAs, small nuclear RNAs (snRNAs), small nucleolar RNAs (snoRNA), lncRNAs, and piwi-interfering RNAs (piRNAs), were detected in human biofluids. Approximately 19.043%, 3.39%, 1.89%, 1.20%, and 0.40% of human breast milk components were made up by miRNAs, snoRNAs, lncRNAs, piRNAs, and snRNAs, respectively [56].

Among noncoding RNAs, miRNAs are the most important in human breast milk exosomes. Therefore, in this article, more detailed information was provided about human breast milk miRNAs.

##### miRNAs

**Figure 2:**
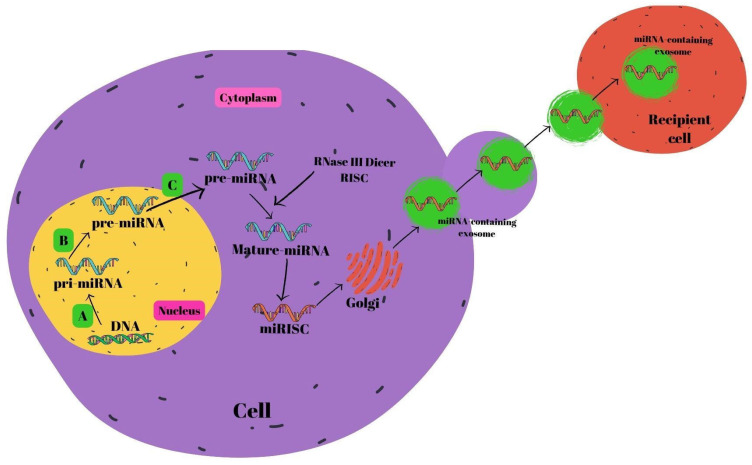
Schematic summary of miRNA biogenesis. (A) RNA polymerase II, (B) DiGeorge syndrome critical region 8 gene (DGCR8) and RNase III Drosha, and (C) RNase III Dicer. Abbreviations: miRNA: micro ribonucleic acid, RISC: RNA-inducing silencing complex, DNA: deoxyribonucleic acid.

miRNA biosynthesis begins in the nucleus [57]. The endogenous coding process of miRNAs occurs in the genome. First, pri-miRNAs are formed from DNAs with the help of RNA polymerase II. RNase III Drosha and the DiGeorge syndrome critical region 8 gene (DGCR8) pair to generate pre-miRNAs. RAS-related nuclear protein (RAN) binds GTP, forming a complex with exportin-5 that transfers the resultant pre-miRNAs into the cytoplasm. Pre-miRNAs join with the RNA-inducing silencing complex (RISC) in the cytoplasm to generate mature miRNAs mediated by RNase III Dicer [58,59,60]. Mature miRNAs are subsequently secreted out of the cell by the Golgi, allowing them to pass into the systemic circulation [61], or the P-body fuses with late endosomes which also contain mature miRNAs. After this combination, miRNA-containing exosomes are released. Exosomes that are released and reach the relevant cells are received by the recipient cells within the framework of receptor–ligand interactions [62]. The miRNA biogenesis is shown in Figure 2.

miRNAs are abundant noncoding RNAs that regulate gene expression by destabilizing target mRNAs and inhibiting translation. They interact with target mRNAs through mechanisms such as cotargeting, degradation, and interactions with RNA-binding proteins [63]. miRNAs exhibit cooperativity in gene regulation, where a single miRNA can target multiple genes and a single gene can be regulated by multiple miRNAs. They are estimated to negatively regulate at least 30% of human genes, impacting various biological processes such as development, immune responses, apoptosis, and cancer [64]. Recent studies have extensively examined the regulation of miRNAs due to their involvement in biological processes and their contribution to the development and progression of various human diseases, such as several types of cancer, retinal disorders, autoimmune diseases, neurodegenerative diseases, and cardiovascular and kidney diseases [65,66,67,68,69,70,71,72].

A systematic review by Tingö et al. [73] revealed that miRNA-148a-3p, miR-NA-30a/d-5p, miRNA-22-3p, miRNA-146b-5p, miRNA-200a/c-3p, and the 5p end of let-7 were the top ten miRNA species noted in the lipid, cellular, and skim milk fractions of human breast milk [73]. A study by Melnik et al. [11] showed that hsa-miRNA-148a-3p is the most dominant human breast milk exosomal miRNA [11]. Another study confirmed that in different fractions of human breast milk, including fat, whey, and extracellular vesicles, miRNA-148a-3p is the most abundant miRNA [74]. A study by Golan-Gerstl et al. [75] identified ten miRNAs that were highly expressed in human breast milk. Accordingly, the ten most abundant miRNA species in skim milk included miR-NA-148a-3p, miRNA-30a-5p, miRNA-99a-5p, miRNA-6073, miRNA-146b-5p, miR-NA-200a, miRNA-21-5p, miRNA-30d, let-7b-5p, and let-7a-5p. However, unlike skim milk, miRNA-184, miRNA-378-3p, miRNA-320-3p, and miRNA-22-3p were among the ten most abundant miRNA types in human breast milk fat [75]. Leiferman et al. [76] reported ten different miRNA types in human breast milk, including let-7f-5p, miR-NA-30d-5p, let-7a-5p, miRNA-146b-5p, miRNA-125a-5p, let-7b-5p, miRNA-21-5p, let-7g-5p, miRNA-423-5p, and miRNA-30a-5p [76]. Reif et al. [77] detected similar miRNA types (miRNA-148 and miRNA-320) in human breast milk exosomes [77]. A study by Lifei et al. [78] noted that milk exosomal miRNAs were rich in miRNA-375 and let-7g-5p, as well as miRNA-99a-5p, let-7b-5p, and miRNA-21-5p [78]. In another study, miRNA types hsa-miRNA-200c-3p, hsa-miRNA-148a-3p, hsa-let-7i-5p, hsa-miRNA-146b-5p, hsa-miRNA-200a-3p, hsa-miRNA-30a-5p, hsa-miRNA-21-5p, hsa-miRNA-26a-5p, hsa-let-7f-5p, and hsa-miRNA-146a-5p showed their presence from high to low levels, respectively [56].

## 3. Factors Affecting Human Breast Milk Exosomes

Although the mechanisms leading to the alterations in exosome composition in human breast milk have not yet been clearly explained, studies showing that the preterm or term birth of an infant, mode of delivery, lactation period, human breast milk storage conditions, heat treatment, digestion, maternal nutrition, stress during pregnancy, body weight, and maternal chronic diseases affect the exosome composition in human breast milk are rapidly increasing. Also, human breast milk exosomes are related to cell origin, function, and communication.

### 3.1. Newborn-Associated Factors

#### 3.1.1. Lactation Period

The breast milk composition of bioactive compounds dynamically varies during different stages [7]. The lactation stage affects miRNA concentrations in human breast milk [25]. Milk collected on 3–8 postnatal days had higher exosome concentrations than mature milk collected in the second month [18]. Similarly, in another study, colostrum had higher miRNA concentrations than mature milk, [79]; a study by Xi et al. [80] revealed that colostrum had lower miRNA-30B levels and greater let-7a and miRNA-378 concentrations than mature milk [80]. A study by Shiff et al. [81] compared human breast milk taken from the mothers of premature infants with that of mothers who delivered term infants. The results showed that compared with term human milk, miRNA-320a is secreted less in preterm human milk, whereas miRNA-148a is secreted more [81]. A study conducted by Freiría-Martínez et al. [82] examined term mature milk, term colostrum, moderate/very preterm mature milk, and moderate/very preterm colostrum. In the same study, term mature milk had higher levels of miRNA-16-5p, miRNA-146a-5p, miRNA-20a-5p, and miRNA-17-5p than moderate/very preterm mature milk. Comparing between term mature milk and term colostrum showed that miRNA-141-3p and miRNA-200c levels were higher in term colostrum. Moderate/very preterm colostrum had higher miR-NA-125b-5p, miRNA-29a/b/c, and miRNA-106b-5p levels than term colostrum [82]. In a different study, human breast milk samples from nursing mothers in the second, fourth, and sixth months following birth showed a total of 1195 mature miRNAs. Notably, one-third of the miRNAs were expressed differently and their levels significantly in-creased in the fourth month of lactation, despite the fact that the total miRNA concentration did not change during the first 6 months of lactation [6,83]. Furthermore, differences were observed in the expression of several species and miRNAs between fore- and hind-milk [21].

Studies examining noncoding RNAs at different lactation stages have been conduct-ed. A study by Yan et al. [84] examined preterm human breast milk and term human breast milk. A total of 44 different lcRNAs were identified in human breast milk from both types [84]. A study by Mourtzi et al. [85] examined the milk of mothers who delivered at term or prematurely in the third week; the results showed that mothers who delivered their babies on time had higher lncRNA concentrations than those who did not [85].

#### 3.1.2. Preterm/Term Birth

Preterm or term birth affects a newborn’s exosome formation and the immunological functions of exosomes [32]. The hormonal profile and miRNA concentration of the human breast milk of mothers with preterm infants change [6]. To shield proteins, miRNAs, and mRNAs from freeze–thaw cycles, acidity, and ribonuclease digestion, exosomes encapsulate them in a phospholipid membrane [86]. In this manner, exosomes can reach the newborn’s intestinal lumen, be absorbed, and fulfill their functions without affecting their contents. Mothers who deliver preterm infants have lower prolactin, estrogen, and progesterone concentrations in their milk. Affecting miRNAs may enable the premature infant to benefit more from exosomal content by affecting mechanisms, including the regulation of adipogenesis, glucose homeostasis, and B-cell proliferation [6,79]. Both fat-free and lipid fractions of preterm colostrum samples had higher miRNA-148 levels and lower miRNA-320 levels than those of term colostrum samples [25,81].

In a different study, the samples of human breast milk collected 3–4 weeks following birth from both term and preterm babies were examined. The expression of nine miRNAs (miRNA-378a-3p, miRNA-378c, miRNA-378g, miRNA-1260a, miRNA-1260b, miR-NA-4783-5p, miRNA-4784, miRNA-5787, and miRNA-7975) involved in metabolic processes such as elemental metabolism and lipid biosynthesis were different in the lipid and skimmed milk fractions. The most significant increase in preterm human breast milk miRNA composition affects glycosphingolipid biosynthesis, which is vital for neurodevelopment. Moreover, in the lipid fractions of preterm human breast milk, 45 and 68 miRNAs were upregulated and downregulated, respectively [6,25,87]. miRNA-148a affects the amount of food consumed by modulating the hypothalamic cholecystokinin receptor 2 (CCK2R or CCKBR) via the melanocortin, opioid, and dopaminergic systems that are associated with food intake in mice [6,88]. Similarly, Kahn et al. [89] examined the human breast milk of term and preterm infants, and noted alterations in miRNA composition and that extremely preterm babies had higher expression levels of miRNA-22, particularly miRNA-148a. Furthermore, a difference in the expression of 21 low-abundance miRNAs was observed between preterm and early-term infants; however, the abundant miRNAs in preterm infants were comparable to those in term infants [11,89]. Zhou et al. [90] detected 6756 circRNAs in the colostrum samples of mothers with both term and preterm infants. In preterm colostrum, 66 of these circRNAs were upregulated and 42 downregulated. In both term and preterm colostrum, exosomes stimulate the expression of vascular endothelial growth factor and the proliferation of small-intestine epithelial cells [90].

In a different study wherein the milk of preterm and term mothers was evaluated, 88 lncRNAs were identified in human breast milk exosomes. More than 85% of human breast milk samples contained 13 lncRNAs, more than 50% contained 31 lncRNAs, and at least twice as many lncRNAs were noted in the human breast milk of term babies. It has been interpreted that the lncRNA changes in human breast milk exosomes are compatible with the adaptive response to preterm hypoxic conditions [85]. A lncRNA that takes part in the DNA damage response and fixes pathways is called noncoding RNA activated at DNA damage (NORAD). Owing to its ability to guard against oxidative stress, apoptosis, and inflammation caused by cerebral ischemia, it has been dubbed “the guardian of the human genome” [90]. Although NORAD was detected in human breast milk that was either preterm or term, its expression was markedly downregulated. The lncRNAs of human breast milk have been the subject of less research than miRNAs; however, the association of these exosomes with neurodevelopment requires more research [25,85].

Using proteomics methods, Wang et al. [54] evaluated the milk of mothers who had preterm and term infants, and showed that 47 peptides were upregulated and 23 peptides were downregulated in the milk of mothers who had term infants. Studies have reported that these peptides are involved in immunological responses, metabolic processes, bio-logical adhesion, cell division, and proliferation. In the necrotizing enterocolitis (NEC) animal model, extracellular vesicles in the human breast milk of the preterm infant were observed to protect villus integrity against damage and repair erythrocyte proliferation compared with untreated NEC-like mice [21,54].

#### 3.1.3. Storage Conditions and Heat Treatment

Donor human breast milk banks are used in cases wherein the mothers of preterm infants cannot produce sufficient milk. Donor milk must undergo pasteurization before use [17]. Holder pasteurization (HoP) (62.5 °C for 30 min) is generally applied for donor human breast milk [91]. Exosomal membranes and contents are influenced by this process, which reduces them by approximately 50% and keeps infants from experiencing the protective benefits of exosomal contents [17,92]. Additionally, in human breast milk samples collected on the 50th day of lactation, the effects of high-pressure processing (HPP) and homology parsing (HoP) on miRNAs were examined. Comparing the milk’s miRNA concentration to the untreated control samples revealed that HPP caused a minor decrease. Conversely, HoP caused an 82-fold decrease in the whole sample and a 302-fold decrease in exosomes, leaving insufficient reads for further analysis [93]. Changes in miRNA composition before and after HPP were destabilized under high pressure and miRNA-29 was sensitive to HPP, whereas miRNA-30d-5p was reasonably stable. The study’s findings indicate that HPP damages human milk miRNAs more than HoP and that further investigation is required into the processing techniques employed in human milk banks [11,93].

Human breast milk exosomes are also affected by the storage process. Another study that examines this effect observed that after 4 weeks of storage at 4 °C, the number of exosome-sized vesicles in human breast milk gradually decreased, reaching 49% ± 13% of fresh milk samples. Exosome losses at week 4 were not statistically significant in frozen and preservative stored samples [76]. Wang et al. [94] analyzed exosomes and miRNAs after human breast milk was frozen at −80 °C and noted that the number of exosomes was approximately 50% of that in fresh human breast milk [94].

The recovery of milk exosomes following heat treatment is related to temperature and time [11]. Milk extracellular vesicles, milk exosomes, and miRNA cargos in commercial cow milk were destroyed by ultra-heat treatment (UHT) and boiling, whereas pasteurization did not change the number of milk extracellular vesicles and retained approximately 25–40% of the total small RNA content [95,96].

It is important to mention that the lyophilization of human breast milk seems to be an optimal storing and transporting approach regarding the quality of exosomes. Lu et al. [97] developed a method that enabled lyophilizing cow milk exosomes, which can be stored at 2–8 °C for 15 months or at room temperature for ca. 3 months [97]. However, the conversion of the method to lyophilize human breast milk exosomes in an economical and high-capacity manner is still warranted.

#### 3.1.4. Digestive System

Milk exosomes prevent damage to miRNAs by enzymes, chemicals, mechanical degradation, and acidic environments resembling the stomach and pancreas [20,98]. The intestinal uptake of miRNA-enriched milk exosomes may be facilitated by inflammatory gut conditions and increased intestinal permeability during the postpartum phase [99]. Exosomes penetrate human intestinal crypt-like cells by identifying 288 mature miRNAs. In a different investigation that simulated gastropancreatic digestion, hsa-miNA-22-3p was the most prevalent miRNA. As the overall abundance of miRNAs in human breast milk exosomes is stable following digestion, thus absorption from the intestine is possible [53].

Preterm newborns’ intestinal epithelial cells (IECs) contained chemicals unique to cows after nine days of colostrum or cow formula feeding. Studies have reported that colostrum supplementation with cel-miRNA-39-5p/-3p increases the levels of “argonaute RISC catalytic component 2 (AGO2)” and cel-miRNA-39-3p in piglet blood. Milk-derived miRNAs were absorbed and passed through the digestive tracts of both newborn porcine and humans [100].

### 3.2. Maternal-Associated Factors

Conditions associated with maternal health affect the nutritional content and bioactive components of human breast milk, as mentioned above. Changes in the functional cargo of human breast milk exosomes shaped by these maternal conditions also shape health outcomes across generations [101]. 

#### 3.2.1. Cesarean Section

Mothers who give birth by cesarean section have different miRNA expression levels in their milk. The increase in exogenous oxytocin levels during vaginal birth is responsible for the increase in miRNA-148a and miRNA-30 levels, and the decrease in miRNA-320 levels in human colostrum [102]. Additionally, in the colostrum of mothers who did not receive exogenous oxytocin, miRNA-320 was expressed more than miRNA-148a. This finding has been linked to a higher risk of type 2 diabetes mellitus (T2DM) in later life by upsetting the cesarean section miRNA-148a/miRNA-320 signaling balance [99]. In contrast to vaginal births, mothers who had cesarean sections had considerably lower miR-NA-148a and miRNA-125b levels in their mature human breast milk and during the transitional phase [103]. Moreover, the frequency of exclusive breastfeeding decreases after birth by cesarean section. Considering the reduced frequency of exclusive breastfeeding following a cesarean section, these changes in the exosome content of human breast milk may adversely affect the epigenetic programming of infants in the postnatal period.

#### 3.2.2. Maternal Nutrition

Several studies on the effect of maternal nutritional characteristics, especially micro-nutrients, on maternal biochemical parameters and their levels in human breast milk have been conducted [28,104,105]. However, research on how maternal nutrition shapes hu-man breast milk exosomes, which is a current research topic, is very limited. Future re-search in this area will emphasize the significance of maternal nutrition and its impact on the quality of human breast milk, in addition to the fact that human breast milk is a vital nutritional source for newborns.

Certain miRNA levels in the lipid fractions of human breast milk are influenced by maternal diet. Women who had high-fat or high-carbohydrate diets with similar energy and protein had higher miRNA-67 and miRNA-27 expressions [104]. A study by Lukasik et al. [105] reported that of five different miRNA species of plant origin (miRNA 166a, miRNA-156a, miRNA-157a, miRNA-172a, and miRNA-168a), only miRNA-168a and miRNA-156a were detected in human breast milk exosomes [105]. Studies have reported that miRNA-148a-5p and miRNA-146b-5p are associated with maternal weight, and miRNA-26a-5p is related to the lipid milk fraction [80,83,106]. Although data on the mechanisms are limited, studies have suggested that some miRNAs in human breast milk affect maternal weight and infant body composition [106,107].

Along with human studies, in animals fed the obesogenic diet model, compared with the control group, miRNA-222 levels increased, whereas miRNA-200 and miRNA-26 levels decreased [108].

#### 3.2.3. Maternal Stress

Maternal stress (psychological distress) negatively affects a child’s health, growth, and development [6,21,109]. The relationship between extracellular vesicle-derived miRNAs and maternal stressors such as adverse life events during pregnancy was evaluated in 80 mothers. This increase in maternal stress has been linked to the epigenetic regulation of pathways, such as fatty acid metabolism, steroid biosynthesis, and the Hippo signaling pathway, which control organ growth [110]. It is believed that further studies on the interaction between maternal stress and extracellular vesicle-derived miRNAs will provide a clearer demonstration of this relationship [21].

#### 3.2.4. Maternal Overweight and Obesity

There is a positive association between the overweight or obesity of mothers and their children, reflected also in human breast milk contents [111]. In the first month of lactation, Shah et al. [112] assessed miRNA-148a and miRNA-30b levels in the milk of 30 women with normal weight and 30 women with overweight/obesity, and discovered that the levels were lower in mothers with overweight/obesity. After adjustment for birth weight, gender, and gestational age, these miRNAs were substantially linked to infants’ anthropometric measurements, and each unit decrease in miRNA-148a levels led to a 0.6 kg increase in body weight and a 0.3 kg increase in fat mass. This significant relationship disappeared between 3 and 6 months of lactation [112]. miRNA-148a and miRNA-30b can stimulate the expression of uncoupling protein 1 (UCP-1), a major inducer of thermogenesis that converts white to beige/brown adipose tissue. Moreover, miRNA-22, miRNA-148a, and miRNA-30b are believed to have anti-inflammatory effects and suppress nuclear factor-κappa B (NF-κB) and interleukin (IL)-6 expressions [11].

Zamanillo et al. [106] examined milk samples from 21 mothers with overweight/obesity and 38 healthy mothers with normal weight, and the growth of their babies up to 2 years old [106].

It has been reported that mothers with a normal body weight had lower leptin, adiponectin, and partly different miRNA levels than mothers with overweight/obesity. An inverse relationship was reported between the expression of leptin, adiponectin, miRNA-17, miRNA-103, miRNA-181a, miRNA-let7c, miRNA-222, and miRNA-146b in the milk of mothers with nor-mal weight and the body mass index (BMI) of their infants [106]. Another study discovered that maternal BMI was negatively correlated with the expression of most miRNAs examined in the study (374 of 419) in human milk extracellular vesicles [113]. Cho et al. [114] analyzed exosomes in the milk of obese mothers and reported changes in 19 miRNAs, including miRNA-575, miRNA-630, miRNA-642a-3p, and miRNA-652-5p, related with neurological diseases and psychological disorders [114].

#### 3.2.5. Maternal Chronic Diseases

The miRNA cargo of human breast milk exosomes is affected by gestational diabetes mellitus (GDM). Shah et al. [115] analyzed the milk of 32 mothers with GDM and 62 mothers without GDM, as well as the growth and body composition of their infants in the first 6 months. The milk of mothers with GDM had lower miRNA-148a, miRNA-30b, miRNA-let-7a, and miRNA-let-7d levels than the milk of healthy mothers, correlated positively with maternal obesity [112,115]. In the first month of life, the weight and fat mass of infants were positively correlated with miRNA-30b levels and negatively correlated with miRNA-148a levels [32]. However, according to the study of Chutipongtanate et al., miR-NAs in the human breast milk of women with type 1 diabetes mellitus (T1DM) did not in-crease infants’ risk of developing T1DM or an inflammatory disease [6,21]. Exosomes from the milk of mothers with GDM and healthy mothers showed different regulatory bioactivities both in HepG2 cell cultures and the liver of Balb/c mice in vivo [116]. By binding to the 3′ untranslated regions (3′-UTR) of mRNAs, miRNA-101-3p in exosomes in the milk of mothers with GDM suppresses its target gene mechanistic target of rapamycin (mTOR) [107,117].

Mothers with T1DM showed altered levels of several miRNAs in their milk samples. Mirza et al. [101] reported that the milk of mothers with T1DM (*n* = 26) had higher im-munemodulating miRNA levels than that of healthy mothers (*n* = 26). In a study in which 631 miRNAs were identified, the expression of six human milk exosome-derived miRNAs increased in the milk of mothers with T1DM, whereas that of three decreased. These miRNAs have been shown to influence proinflammatory cytokine production through PI3K/AKT, which in turn regulates the immune response and cell cycle [101]. Moreover, it has been shown that a significant increase in miRNA-4497 and miRNA-3178 levels may increase the expression of tumor necrosis factor-alpha (TNF-α), a proinflammatory cytokine, from THP1-transfected monocytes in vitro [101,118].

Evidence exists that human breast milk reduces the risk of developing T2DM later in life [119]. The number of studies on the relationship between cow milk consumption and T2DM is also increasing. In rodent models, pancreatic cells have been shown to be immature during lactation and proliferate by adenosine monophosphate-activated protein kinase (AMPK) activity suppression and mTOR complex 1 (mTORC1) pathway activation. Weaning initiates a process wherein insulin secretion and mTORC1 activity decrease, AMPK activity increases, and β cells metabolically proliferate because of glucose stimulation. The termination of the uptake of miRNA-148a, an AMPK inhibitor, phosphatase and tensin homolog (PTEN), and mTORC1 suppressor from human breast milk is associated with this metabolic change. Cow milk miRNA-148a induces an immature mTORC1–high/AMPK–low gene expression profile in the cell, resulting in impaired insulin secretion, increased mTORC1-driven endoplasmic reticulum stress, decreased autophagy, and early cell apoptosis. Cow milk miRNAs are inactivated in infant formulas by boiling, UHT, and bacterial fermentation, but not pasteurization [99]. Further research on the effects of pasteurized cow-milk-derived miRNAs on T2DM pathogenesis is needed [6]. Table 1 presents the summary of maternal-associated factors affecting human breast milk exosomes.

## 4. Possible Health Effects of Human Breast Milk Exosomes

The present review explains the possible effects of exosomal components in human breast milk on the immune system, cardiometabolic system, cancer, and NEC.

### 4.1. Effects on the Immune System

While the adaptive immune system develops in newborns, the epithelial barrier of the gastrointestinal tract continues to grow and mature. In this instance, human breast milk components are relevant [120]. Immunologic proteins (lactoferrin, α-lactalbumin, secretory immunoglobulin A [sIgA], and lysozyme), cytokines, and oligosaccharides in hu-man breast milk affect immunity. Furthermore, the development, differentiation, metabolism, proliferation, and death of cells and tissues may be influenced by miRNAs, short, and noncoding RNAs noted in human breast milk [15,121]. Studies have identified many miRNAs associated with the immune system in human breast milk [10,98,101,122].

#### 4.1.1. Cell Proliferation and Inflammation

Human breast milk exosomes have been implicated in cell proliferation and inflammation regulation. Studies examining the effects of human milk on cell proliferation have mostly been performed on epithelial cells [54,123]. Preterm human breast milk exosomes significantly promoted normal human epithelial cell proliferation and increased migration (compared with term human milk exosomes). Furthermore, preterm human breast milk exosomes preserved ileal villous integrity and restored the reduction in enterocyte proliferation in rats with NEC. Moreover, peptides isolated from preterm human breast milk exosomes can regulate intestinal epithelial tissue [54]. A study on NEC reported that preterm and term human breast milk exosomes significantly increased epithelial proliferation and migration in vitro [123]. Human breast milk exosomes increased collagen type 1 expression and epithelial cell proliferation in normal epithelial cells. Hence, the afore-mentioned most important miRNA-148a in human breast milk exosomes was effective in PTEN and DNA methyltransferase 1 (DNMT1) regulation in mouse embryonic fibroblasts (MEFs) [124]. Also, miRNA-22 3p (miRNA-22), commonly noted in human breast milk exosomes, promoted cell cycle progression, cell growth, and proliferation in human IECs. In particular, miRNA-22 regulated cell proliferation through CCAAT/enhancer-binding protein δ (C/EBPδ) gene expression inhibition [125].

Human breast milk exosomes improved hyperoxia-induced collapse in lung tissue structure and alveolar number reduction [126]. Human breast milk exosomes inhibited hyperoxia-induced alveolar type (AT)-II cell apoptosis, decreased Fas-associated protein with death domain (FADD) expression, and downregulated C-caspase-3 and C-caspase-9. By inhibiting the IL-17 signaling pathway, human breast milk exosomes downregulated cell apoptosis [126].

Chen et al. [123] revealed 395 lipids in preterm and term human-milk-derived exosomes. Of these, 10 showed a significantly different profile in term and preterm human milk. Moreover, they identified 15 lipid subclasses, including phosphatidylcholine (PC), phosphatidyl serine, and phosphatidyl ethanolamine in preterm and term human breast milk exosomes. It was suggested that 50 of these lipids can regulate IEC function via the extracellular signal-regulated kinase/mitogen-activated protein kinase (ERK/MAPK) pathway. Lipids obtained from human breast milk exosomes showed an effect on the re-duction in lipopolysaccharide (LPS)-induced p-ERK expression [123].

circRNAs, a human breast milk exosome component, have also been shown to have effects on intestinal development by regulating miRNAs. Moreover, by affecting the vascular endothelial growth factor (VEGF) pathway, they affected small IEC proliferation and migration regulation [90].

Human breast milk exosomes have been shown to reduce inflammation and oxidative stress [40,91]. Additionally, they protected against hydrogen peroxide (H_2_O_2_)-induced oxidative damage in IECs [40]. Human breast milk exosomes are able to mitigate the inflammatory cytokine expression of IL-1β, TNF-α, and IL-6 [127]. Miyake et al. [91] showed that pasteurized and crude human breast milk exosomes reduced IL-6 mRNA expression in hypoxia and LPS-induced intestinal organoid injury. Furthermore, raw and pasteurized human breast milk exosomes reduced the increased IL-6 mRNA expression and myeloperoxidase (MPO) expression in NEC, indicating an inflammation-reducing effect [91]. Evaluating exosomes isolated from colostrum, transitional, and mature milk revealed that an LPS-induced increase in TNF-α, Toll-like receptor (TLR) 4, Ki67, and stem cell marker Lgr 5 (leucine-rich repeat-containing G-protein-coupled receptor 5) levels in intestinal organoids were decreased owing to the intervention. The greatest decrease occurred owing to intervention with isolated colostrum milk exosomes. Additionally, the LPS-caused decrease in organoid size reversed [128]. Human breast milk exosomes reduced IL-1β and TNF-α levels in intestinal tissues. Increased transcription and expression levels of intestinal tight junction (TJ) proteins zonula occludens-1 (ZO-1), occludin, and claudin 1 were noted [129]. Human-milk-exosome-derived miRNA-148a 3p reduced NF-Kβ translocation, decreased proinflammatory cytokines IL-6 and TNF-α, and increased anti-inflammatory EgF and IL-10 levels. Through the in vitro regulation of p53 and sirtuin 1 (SIRT1), LPS-induced cell damage was reduced [130].

#### 4.1.2. Immunomodulatory Function

Human-milk-derived exosomes have been shown to have immunomodulatory activity [19,131]. Moreover, innate and acquired immune system development and pathogenic infections are influenced by these exosomes and their components (miRNAs and proteins) [10,132,133,134].

Human breast milk exosomes decreased CD3-induced IL-2, interferon (IFN)-γ pro-duction, and TNF-α, levels as well as increased IL-5 levels in peripheral blood mononuclear cells. Additionally, they increased the number of Foxp3^+^CD4^+^CD25^+^ T regulatory cells [19]. A study by Näslund et al. [131] reported that human breast milk exosomes sup-pressed human immunodeficiency virus type 1 (HIV–1) infection in monocyte-derived dendritic cells and reduced the transmission of infection to CD4^+^ T cells [131]. Colostrum, transitional, and mature milk-derived exosomes reduced the LPS-induced TLR-4 increase in intestinal organoids [128].

Of the miRNAs produced from human breast milk, about 65% are associated with immunological activity [134]. It has been revealed that miRNAs in breast milk have roles in innate and adaptive immunological responses [79]. miRNA-148a-3p, miRNA-146b-5p, miRNA-200a-3p, miRNA-155-5p, miRNA-150, miRNA-181a, miRNA-30b 5p, miR-NA-28a 3p, and miRNA-182 5p in human milk exosomes have been previously identified to have immunomodulatory activities [132,133,134]. Kosaka et al. [98] showed that miRNA-181a and miRNA-155, which are involved in B-cell differentiation, were observed in high concentrations in human milk in the first 6 months. Furthermore, miRNA-92 and miRNA-17 were detected at higher concentrations within the first 6 months of human milk than those in the next 6 months [98].

These miRNAs are involved in normal B-cell development; the promotion of IgM formation; T-cell activation; the promotion of T-helper (Th) 1, Th2, and Th17 responses; the control of IL-12-mediated immune responses; and lymphomagenesis [135,136,137]. Higher circulating regulatory T cell (Treg) levels have been linked to higher miRNA-148a-3p and let 7d-3p levels in human milk [138]. In human IECs, miRNA-22, another miRNA that is frequently present in human milk, has been shown to play several roles, including the promotion of cell cycle progression, cell growth, and proliferation; protection against viral infection; type 1 interferon signaling pathway regulation; immune function production and regulation; and apoptosis inhibition [125]. miRNAs isolated from human milk are involved in the fc-epsilon receptor signaling pathway, TLR signaling pathways, innate immune response, T-cell-related pathways, and pathogenic infections. Exosomal proteins are involved in 31 different immune processes, including defense response and immunity, phagocytosis and complement activation, immunoglobulin and antigen receptor-mediated processes, cytokine regulation and response, innate immune response and inflammation, and cell migration and activation. The following three key proteins are associated with pathogenic microbial infections: intercellular adhesion molecule 1 (ICAM-1), TLR2, and fibronectin 1. These proteins are vital in mediating the proinflammatory responses required for managing infections and facilitating the elimination of microorganisms [10]. In summary, human-breast-milk-derived miRNAs are promoting innate and acquired immune system development.

Human-milk-derived extracellular vehicle miRNAs exerted their effects on the immune system by affecting the AMPK signaling pathway, NF-κB signaling pathway, IL-15 production, T-cell receptor signaling pathway, and signal transducer and activator of transcription (STAT)-3 pathway involved in immune response, cell growth, and survival. The expressions of IL-10, an anti-inflammatory cytokine, and IL-15, which is controlled by IFN-γ, were increased [139]. Of note, one of the components of human milk extracellular vehicle, proteins, may also interact with cell types in the oral mucosa to regulate the epithelial barrier of newborns and ensure the regular development of the innate and acquired immune systems. Human milk extracellular vehicle enhances the function of the epithelial barrier by promoting cell migration through p38 MAPK and cytoskeleton reorganization. Moreover, extracellular vehicle proteins may interact with several signaling pathways and affect TLR response modulation, and T-cell activation and differentiation [120].

The expression of 41 miRNAs in the exosomes of HIV-1-infected mothers was different compared with that of the control group. Particularly, 13 miRNA types were upregulated, including miRNA-148a-3p, miRNA-320e, miRNA-630, miRNA-378g, and miRNA-23a-3p. This finding suggested that HIV-1 infection can potentially influence exosome composition [140].

### 4.2. Effects on Cancer

Human breast milk’s chemopreventive properties in pediatric lymphoma and leukemia have been thoroughly investigated [141,142,143]. According to a meta-analysis by Fan et al., breastfeeding may have a protective effect on maternal breast cancer, ovarian cancer, and childhood leukemia [144]. In another systematic review and meta-analysis, breast-feeding was associated with a reduced risk of childhood Hodgkin’s disease, acute lymphoblastic leukemia, and neuroblastoma [145]. Other studies have similarly shown that breastfeeding is associated with a reduced risk of ovarian and breast cancer in mothers [146,147].

Studies evaluating the effects of components of human breast milk exosomes on cancer are limited and have generally been conducted at the cellular level by isolating exosomes from breast milk. Therefore, explaining human breast milk exosomes’ positive/negative mechanisms in relation to all cancer types is difficult [55,124]. Human breast milk exosomes affect the epithelial–mesenchymal transition (EMT), cancer cell miRNAs, some gene expressions, and cell proliferation in some cancer cells [55,124]. Colon cancer cell incubation with human breast milk exosomes caused an increase in miRNA-148a expression. Furthermore, miRNA-148a silencing in tumor cells resulted in increased tumor cell proliferation. Human breast milk exosomes downregulated collagen type I expression in normal epithelial cells, and downregulated twist1 gene, DNMT1, and PTEN protein expressions in normal cells. Human breast milk exosomes have shown a beneficial effect by inducing EMT and cell proliferation, which are crucial for intestinal growth and development. However, these mechanisms were inactive in colon cancer cells [124]. Owing to the incubation of miRNAs isolated from human breast milk exosomes into CRL 1831 cells (human normal intestine cell line), K562 (leukemia cells), cell miRNA-148a folding was upregulated in both cell types, while DNMT1 folding was downregulated. This finding suggests that miRNA-148a can cause epigenetic changes in cells owing to its effect on DNA methylation [75]. It has also been reported that breast milk exosome components may have a negative influence on breast cancer risk. A study on breast cancer reported that high TGF β2 (in human breast milk exosomes) levels promoted EMT in both malignant and benign breast cells by altering cell morphology, actin cytoskeleton, and cell–cell junctional structure; increasing alpha-smooth muscle actin (SMA) and vimentin levels; and decreasing E-cadherin levels [55]. However, it is important to increase studies to elucidate the mechanisms of positive or negative effects of breast milk exosomes and its derived components on cancers.

**Figure 3:**
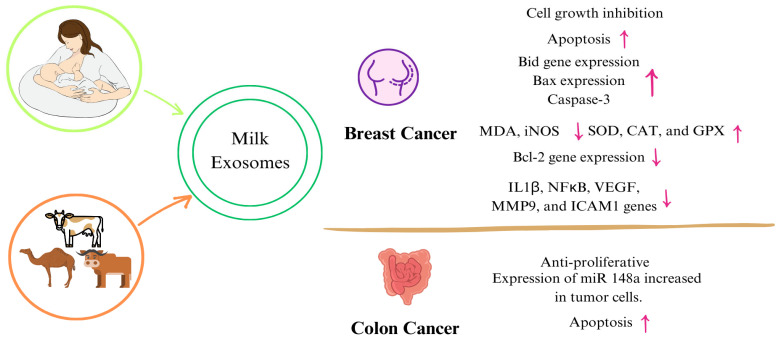
Mechanisms of milk exosomes on certain types of cancer. (↑: increase, ↓: decrease) Abbreviations: miRNA: micro ribonucleic acid, NF-κB: nuclear factor-κappa B, IL-1β: interleukin 1beta, MMP9: matrix metalloproteinase, VEGF: vascular endothelial growth factor, ICAM: intercellular adhesion molecule 1, MDA: malondialdehyde, iNOS: inducible nitric oxide synthase, SOD: superoxide dismutase, CAT: catalase, GPX: glutathione peroxidase.

Evaluating the effects of exosomes obtained from other milk besides human milk on cancer showed that cancer cell proliferation, inflammation, and oxidant activity de-creased, whereas apoptosis, tumor DNA damage, and antioxidant activity increased [148,149,150]. Table 2 and Figure 3 are a summary of the studies on the mechanisms of exosomes and their components obtained from both human and animal milk exosomes on cancer. Although studies evaluating the impact of miRNAs on cancer have been conducted [151,152,153], this section only focuses on studies involving milk exosomes and milk-derived components. In line with these studies, although it is thought that exosomes passed from breast milk to the infant may have positive effects on reducing the risk of cancer formation, studies on exosomes and their components isolated from breast milk are limited. Increasing animal and human studies in addition to cellular studies in cancer will help elucidate the mechanisms.

### 4.3. Effects on Cardiometabolic Diseases

#### 4.3.1. Effects on Obesity

Obesity, which is characterized as a “pandemic” owing to its increasing global prevalence, is a complex disease wherein various factors including age, gender, genetics, physical activity level, socioeconomic factors, and birth weight play a role in its etiology [155]. Prenatal and neonatal periods also play a significant role in obesity etiology [112,156]. Several research groups from different countries have investigated the contribution of neonatal nutrition to the risk of obesity in childhood and adolescence [155,157,158,159]. These studies revealed that breastfed infants had a lower risk of developing obesity in later periods than those fed with infant formulas, whereas the risk of obesity significantly decreased with increasing breastfeeding duration [155,157,158,159]. These protective effects of human milk against obesity are associated with its various properties, including the containing hormones that regulate energy metabolism such as leptin and adiponectin, its energy and nutrient content being at a level that can meet the needs of infants, and the containing probiotic microorganisms such as Lactobacillus and Bifidobacterium, and oligosaccharides with prebiotic properties [155]. Recent studies have shown that the exosomes contained in human milk are also responsible for the protective effect against obesity.

Milk exosomes contain proteins (CD9, CD63, CD81, CD82, HSP70, HSP90, Alix, TSG101, TGF-β, annexin, and Rab GTPases), lipids, mRNAs, miRNAs, and lncRNAs [11]. Human milk plays a regulatory role in several metabolic pathways, especially through its exosomal miRNAs and lncRNAs. Several studies reported that miRNA-148a, miRNA-29a, miRNA-29b, miRNA-32, miRNA-30b, miRNA-30a, miRNA-146b, miRNA-4454, miRNA-494-3p, miRNA-21, miRNA-26a, miRNA-30d, miRNA-181a, miRNA-22, miR-NA-141, miRNA-27b, and let-7a are the most abundant miRNAs in human breast milk exosomes [11,101,112,114,160]. miRNA-148a is reported to cause hypomethylation and increases the expression of genes that play a significant role in growth and development (insulin (*INS*), insulin-like growth factor-1 (*IGF1*), fat mass- and obesity-associated gene (*FTO*), forkhead box protein 3 (FOXP3), nuclear factor erythroid 2-related factor 2 (NRF2), and lactase gene (*LCT*)) by suppressing DNMT1 transcription [161]. In a similar manner to miRNA-148a, miRNA-29, another human milk exosomal miRNA, activates anabolic processes that play a significant role in the growth and development of infants by increasing *INS*, IGF-1/AKT/mTORC1, and *FTO* gene expressions through suppressing DNMTs. The continuous activation of these metabolic pathways may lead to an increase in adipogenesis, lipogenesis, and obesity. However, a negative feedback mechanism in the expression of these miRNAs is observed. For example, DNMT1 overexpression leads to miRNA-148a and miRNA-152 gene hypermethylation. Simultaneously, DNMT1 levels, the direct target of miRNA-148a and miRNA-152, are inhibited by miRNA-148a and miRNA-152 overexpression [162].

The exosomal miRNA content of human milk is characterized according to the physiological needs of the infant. For example, the exosomal miRNA contents of colostrum and mature milk, milk from preterm mothers, and milk from full-term mothers differ [81,163]. The colostrum of mothers with preterm birth had a significantly higher miRNA-148a expression than the colostrum of mothers with full-term birth; the miRNA-320 level of full-term human milk was significantly higher than that of preterm human breast milk. The increase in miRNA-320 expression leads to fatty acid synthase expression suppression, and this effect is opposite to the anabolic effects of miRNA-148a. Consistent with the needs of preterm infants attempting to catch up with growth, it was observed that they have higher miRNA-148a expression, which activates anabolic processes, and lower miRNA-320 expression, which suppresses these processes, in the human breast milk exosomes [81].

The expression of exosomal-milk-derived miRNA is influenced by maternal diet, maternal disease, and maternal lifestyle and stress [163]. A study compared the exosomal markers and miRNAs of milk from 47 mothers with normal body weight before pregnancy and 18 mothers with obesity, and reported that CD326 (EpCaM) was the most expressed marker in both groups, and miRNA-30b-5p, miRNA-4454, miRNA-494-3p, and let-7 were the most abundant miRNAs [114]. The milk of mothers with overweight/obesity had significantly lower exosomal miRNA-148a and miRNA-30b levels than that of mothers with normal weight. Regardless of the BMI group, a 43% decrease in the fold change of miR-NA-148a and a four-fold increase in the fold change of miRNA-30b from 1 to 3 months following lactation were noted [112]. The overexpression of miRNA-30b/c in brown and subcutaneous adipocytes enhances UCP-1 and cell-death-inducing DFFA-like effector A (Cidea) expression, which is crucial for thermogenesis, whereas its inhibition decreases UCP-1 levels. Additionally, miRNA-30b/c targets RIP140, a corepressor of thermogenic genes [164]. This similarity between exosomal miRNAs in the milk of mothers with overweight/obesity and those with normal weight, and the differences between exosomal miRNAs (miRNA-148a and miRNA-30b) in the milk at the first month following delivery are believed to be the protective mechanisms against obesity in the infants of mothers with overweight/obesity.

In summary, current studies examining the effect of human breast milk exosomes on the development of obesity in infants have focused on the activity of exosomal miRNAs. The results obtained from these studies showed that human milk exosomal miRNAs vary according to the needs of the infant (preterm and term; infant age, 1–3 months) and the maternal BMI value to achieve growth or prevent obesity. Thus, as breastfed children are exposed to exosomal miRNAs, it can be assumed that they are protected against obesity better than formula-fed newborns.

#### 4.3.2. Effects on Diabetes Mellitus

DM is mainly characterized by hyperglycemia due to abnormalities in insulin secretion or insulin action [165]. A decrease in the function and number of pancreatic beta cells, which are responsible for insulin production and secretion, is a significant factor in the pathophysiology of T1DM and T2DM [166]. Genes involved in regulating the cell cycle are suggested to impact the β cell mass during development. In humans, β cell replication is crucial during infancy, while it occurs rarely in adults [167]. Therefore, ensuring healthy beta cell development and proliferation during infancy is lowering the risk of diabetes onset in later life. β cell dysfunction or loss can be caused individually or in combination with intrinsic defects, resulting from genetic mutations, extrinsic metabolic stressors such as high systemic insulin demand, and other harmful factors such as autoimmune antibodies, glucolipotoxicity, and inflammation [166,168]. Breastfeeding has a protective effect on beta cell function, and reduces T1DM and T2DM risks during later life [169,170].

Human milk, despite its rich composition of nutrients, antibodies (sIgA), and beneficial microbiota, plays a crucial role in providing optimal nutrition, immune protection, and metabolic regulation (antidiabetic and antiobesity) for infants [29]. Furthermore, during the breastfeeding period, milk exosomes and their miRNAs may interact with neonatal β cells as they enter the systemic circulation and tissues [171]. miRNA-148a could trigger pancreatic β-cell differentiation to an immature phenotype by driving AMPK-to-mTORC1 switching [172]. In this pathway, mTORC1 is essential for regulating multiple β-cell biology parameters, including mass, size, and proliferation [171]. During the weaning process, β cells enter the maturation phase with AMPK stimulation and mTORC1 inhibition, shaping the beta cell phenotype of adulthood [99]. Along with miRNA-148a, other human milk exosomal miRNAs (let-7, miRNA-21, miRNA-30d, miRNA-26, miRNA-146a, miRNA-29a, and miRNA-34) are significant regulators in the differentiation and function of pancreatic β cells [173].

In addition to miRNAs, lncRNAs noted in human breast milk exosomes also have various biological effects, including promoting infant growth and development, and adaptation to environmental factors. The most prevalent lncRNA in human breast milk exosomes, growth arrest-specific 5 (GAS5), has been shown to interact with the insulin receptor promoter and increase insulin receptor transcription. Subsequently, this process improved insulin signaling, which enhances adipocytes to absorb glucose [174]. Individuals with diabetes had lower serum levels of GAS5 expression; a GAS5 value of <10 ng/µL is associated with a twelve-fold increased risk of developing diabetes [175].

**Figure 4:**
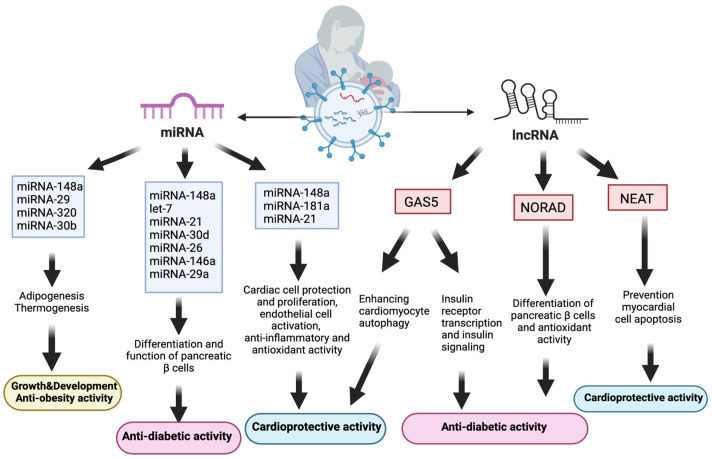
Relationship between human breast milk exosomal components and cardiometabolic diseases. Abbreviations: lncRNAs: long noncoding RNAs, miRNA: micro ribonucleic acid, NORAD: noncoding RNA activated at DNA damage, GAS5: growth arrest-specific 5, NEAT: nuclear paraspeckle assembly transcript 1.

Another lncRNA detected in human breast milk exosomes, NORAD, also interacts with importin-β1 to regulate TGF-β signaling and Smad translocation into the nucleus. The TGF-β/Smad pathway enhances the endocrine-specific transcription factor (Ngn3), thereby leading to the promotion of pancreatic β-cell development [173]. Moreover, NORAD plays a crucial role in infant adaptation to hypoxic conditions following birth. Upregulated during hypoxia, NORAD aids in newborns’ adaptive response to excessive oxygen exposure, which is crucial for preventing oxidative stress. Breastfeeding, containing NORAD in exosomes and antioxidants, is vital for preterm infants’ adaptation to higher oxidative stress levels compared with formula feeding [85]. As oxidative stress and inflammation play a significant role in the pathophysiology of diabetes, infants with NORAD exposure through human milk exosomes are expected to have a lower risk of developing diabetes [176] (Figure 4).

#### 4.3.3. Effects on Cardiovascular Diseases (CVDs)

CVDs are the leading cause of death worldwide, responsible for 17.9 million deaths annually. Cerebrovascular, rheumatic, and coronary heart diseases are among the disorders of the heart and blood vessels that are collectively referred to as CVDs [177]. Coronary artery disease is the most common cause of death worldwide and is characterized by the narrowing or blockage of coronary arteries due to plaque formation within the vessel walls, primarily composed of fatty materials and exacerbated by chronic inflammation. This process restricts blood, oxygen, and nutrient supply to cardiomyocytes, thereby leading to potential complications [178]. Several individual and environmental factors such as hypertension, hypercholesterolemia, obesity, diabetes, age, gender, ethnicity, stress exposure, smoking, genetic propensity, dietary habits, and physical activity level play a role in the pathogenesis of CVDs [179]. In addition to these factors, prenatal and neonatal exposures including nutrition, stress, and environmental pollutants may also affect the CVD susceptibility of infants [180,181,182]. For example, breastfeeding is inversely associated with childhood triglyceride levels at 11 years old, independent of childhood obesity [181]. In another study conducted on this subject, the effect of infant feeding method on adult health indicators was investigated. The results showed that bottle-fed infants had higher plasma glucose levels, low-density lipoprotein (LDL) cholesterol levels, and LDL/high-density lipoprotein ratio at 120 min, whereas exclusive breastfeeding was protective against some CVD risk factors in adulthood; however, systolic blood pressure and BMI were not affected [183]. Human milk possesses potent biological activity, including growth factors, enzymes, antibodies, and stem cells, which are absent in infant formulas. These components play a crucial role in enhancing cardiovascular development in infants [184].

The cardiovascular system relies on diverse cell types such as cardiomyocytes, endothelial cells, and immune cells for its function and maintenance, with their intercellular communication crucial for both homeostasis and disease processes [185]. Exosomes, particularly through paracrine and endocrine functions, potentially mediate this cellular crosstalk in CVDs [185]. Moreover, epigenetic regulators, including miRNAs and lncRNAs, detected in human breast milk exosomes contribute to cardiovascular health maintenance and CVD prevention [186,187,188,189,190]. Cardiac dysfunction, immune dysfunction, and cardio-myocyte apoptosis developed in rats with insufficient miRNA-148 expression; however, these were mitigated by miRNA-148 stimulation. The miRNA-148/pyruvate dehydrogense kinase (PDK) 4/suppressor of mother against decapentaplegic (SMAD) pathway played a crucial role in alleviating myocardial ischemia–reperfusion (IR) injury, and PDK4 knockdown via miRNA-148 expression reversed these effects, restoring cardiac function and immune balance while enhancing antioxidant activity [190]. Additionally, exosomes containing miRNA-21 are efficiently taken up by cardiac stem cells and safeguarding them from apoptosis by decreasing PTEN expression and stimulating the PI3K/AKT signaling pathway. Moreover, these exosomes serve a protective function against oxidative stress-induced cell death [191,192]. miRNA-181a-5p is another human milk exosomal miRNA, and its cardioprotective roles include NF-κB signaling suppression, endothelial cell activation regulation, cell proliferation, and immune cell homeostasis. Specifically, miRNA-181a-5p downregulates TNF-α, which is the key inflammatory response mediator [189].

A major lncRNA in human milk, GAS5, effectively reversed the histopathological alterations caused by diabetic cardiomyopathy (DCM) and improved myocardial function by enhancing cardiomyocyte autophagy. This effect is potentially mediated through a GAS5/miRNA-221-3p/p27 competing endogenous network, highlighting the role of GAS5 in promoting cardiomyocyte autophagy and protecting myocardial function in DCM [186]. In patients with T2DM, DCM progression involves impaired cardiomyocyte autophagy, leading to the accumulation of damaged organelles and harmful metabolites within cardiomyocytes. Consequently, this process contributes to myocardial hypertrophy, fibrosis, and eventual cardiac dysfunction [188]. Therefore, the autophagic effect of GAS5 is highly significant for cardiovascular health protection. Nuclear paraspeckle assembly transcript 1 (NEAT1), another lncRNA in human breast milk exosomes, contributes to cardiovascular health promotion by protecting myocardial cells against apoptosis [187] (Figure 4).

### 4.4. Effects on Intestinal Diseases

Human-milk-derived exosomes offer a potential therapy that could improve the integrity of the early intestine.

Research has indicated that milk exosomes shield exosomal miRNAs from enzymatic, chemical, or mechanical destruction. The stability of bovine milk exosomal miR-NAs for absorption, digestion, and transepithelial transport by IECs has been investigated using an in vitro digestion model. The findings demonstrated that cow milk exosomes can pass through the intestinal barrier and enter the circulation to support cellular activity while shielding miRNAs from digestion processes [193]. In a study of Liao et al. [53], aimed to investigate how simulated gastric and pancreas digestion affects human milk exosome surface markers and gut cell absorption under conditions simulating the infant’s gut. The findings provide new insight into the intricacy and survival of human milk exosome miRNAs throughout pancreatic and stomach simulations, as well as the dynamics throughout the lactation cycle. Exosomes, both digestible and undissolved, enter cells resembling crypts in the human gut. From 24 exosome samples of the intestinal epithelium, 288 mature miRNAs were noted. In this study, hsa-miRNA-22-3p was identified as the most abundant miRNA. It was reported that exosomes protect human intestinal crypt-like cells from oxidative stress [53]. Another study analyzed the potential gastrointestinal transfer of bovine milk miRNAs in neonatal humans and an in vivo pig model. Following enteral feeding via bovine colostrum/formula, the ICs of preterm piglets began to accumulate bovine-specific miRNAs. Piglets supplemented with cel-miRNA-39-5p/-3p in colostrum showed improved blood cel-miRNA-39-3p levels and enhanced AGO2 accumulation in the ICs. The results indicate that in both human and pig neonates, milk-derived miRNAs can transit through the gastrointestinal tract. Milk vertically transfers miRNA signaling to the developing digestive system [100].

Most studies have confirmed the resistance of exosomes to digestion. When exposed to gastric and pancreatic digestive conditions in vitro, milk exosomes prevent miRNA degradation [53,54,100,193,194]. Next, IECs endocytose the milk exosomes and are subsequently moved into the circulatory system [195].

#### 4.4.1. Intestinal Epithelial Function

The selective absorption of food components depends on IECs, which are significant elements of the intestinal barrier. After swallowing, breast milk exosomes can be taken up by IECs by endocytosis and transported to various organs, including the brain, liver, spleen, and heart [41,195,196].

In a different study assessing the impact of rat milk exosomes on rat IECs, treatment with exosomes dramatically enhanced IEC survival, proliferation, and stem cell activity compared with the control. In contrast to milk therapy without exosomes, the study dis-covered that exosomes obtained from milk considerably increased IEC survival and proliferation, and encouraged intestinal stem cell (ISC) activity [17]. These findings provided novel and significant information about how human milk affects IEC growth under nor-mal conditions. Using human colorectal adenocarcinoma epithelial (Caco-2) cells, a study compared the functional role of colostrum with bovine milk at the intestinal interface. The results showed that when Caco-2 cells are co-incubated with other Caco-2 cells, milk exosomes, which are noncytotoxic to human IECs, can preserve metabolic activity and reduce caspase 3 activity. Moreover, the study results suggested that dairy products from cows with different immune response genetics can affect the gut health of humans in different ways [197]. Gao et al. [198] investigated the effect of yak and cow milk exosomal proteins on the mechanism underlying LPS-treated IEC-6 barrier function. The findings demonstrated the effects of yak milk exosomes on therapy to promote epithelial cell barrier formation against intestinal inflammation. Evidence on the relationship between functional dietary components (milk exosome proteins) and PI3K-AKT/C3 signaling in intestinal inflammation, as well as a novel understanding of the processes of milk exosome proteins in alleviating intestinal inflammation and regulating intestinal development were noted. A proteomics study revealed 58 highly expressed and 334 lowly expressed proteins in yak milk exosomes compared with cow milk exosomes. When compared with cow milk exosomes, yak milk exosomes considerably boosted the PI3K/AKT/C3 signaling pathway, thereby lowering the frequency and intensity of intestinal inflammation [198].

Using an ex vivo intestinal organoid model, the study intended to assess the protective effects of exosomes from various stages of human milk production against intestinal damage. In neonatal mice, the protective ability of human breast milk exosomes from different time periods of human breast milk production (colostrum–transitional–mature milk) against intestinal injury were evaluated in vitro and in vivo. When incubated with LPS, human breast milk exosomes could protect against LPS-induced epithelial damage in intestinal organoids, and colostrum showed the best protective effect [128]. Both in vitro and in vivo experiments showed that human milk exosomes encourage IEC expansion and migration [54]. Another in vitro study investigated the effects of human breast milk exosomes on ISC injury and concluded that human breast milk exosomes protected ISCs from H_2_O_2_-induced oxidative stress injury in vitro and that this injury was likely mediated by the Wnt/b-catenin signaling pathway [52]. The results of the study showed that by controlling the VEGF signaling pathway, circRNA_104707 and circRNA_405708 in human breast milk exosomes may be involved in gut health [90]. A study conducted in rats investigated the effects of human breast milk exosomes on IR-induced intestinal damage. At the end of the study, a decrease in intestinal inflammation and an increase in IC proliferation were observed. By lowering mucosal inflammation and encouraging intestinal renewal, human breast milk exosomes can lessen intestinal damage caused by irritants [199].

#### 4.4.2. Necrotizing Enterocolitis (NEC)

NEC is a devastating condition that is frequent in preterm infants [200]. Current treatments for NEC are insufficient, leading to high mortality rates and intestinal function-related short- and long-term complications [201]. Although necrosis slows migration and proliferation, making the host more susceptible to additional injury and facilitating bacterial translocation through exosomes carrying regulatory molecules, restoration entails enterocyte migration from healthy to injured regions. Exosomes are effective in facilitating contact between cells and contain a range of regulatory chemicals, including miRNAs, which are essential for several basic biological functions [202].

Studies have reported that human breast milk exosomes are protective against NEC [17,40,53,91,129]. The lipid particles of human milk contribute to the effects of its biologically active components that affect intestinal growth, barrier function, microvascular development, and immunologic maturation. The lipid particles, especially exosomes, are optimal carriers of bioactive macromolecules, for example, miRNAs. Compared with formula-fed newborns, preterm newborns fed with human milk have a decreased NEC incidence [203,204]. Human milk benefits a developing newborn’s immune system, nutrition, and development through various molecular and cellular pathways.

By developing a NEC model in mice, Hu et al. [205] investigated the effects of human amniotic fluid stem cell (AFSC) exosomes and human breast milk exosomes in vivo and in vitro. In vivo, following human milk exosome initiation significantly restored the number of ileal crypts compared with AFSC exosome intervention. In vitro, human breast milk exosomes selectively reduced IECs’ inflammatory response, whereas AFSC exosomes selectively controlled IEC migration [205]. Pisano et al. [206] conducted a study in rats to determine if exosomes made from human milk can block NEC. This study demonstrated that human breast milk exosomes were efficient in suppressing experimental NEC in a mouse model that has undergone thorough validation. Furthermore, it demonstrated that human-milk-derived exosomes have anti-apoptotic and pro-proliferative implications for IECs [206].

Human-milk-derived exosomes can prevent IECs from cell death by protecting hu-man intestinal crypt-like cells from oxidative stress [40,53]. H_2_O_2_-induced oxidative stress caused a 50% loss in viable cells. Studies on human breast milk exosomes promoting gastrointestinal and immune system development through barrier function, pathological microbial luminal sensing, and antimicrobial peptide upregulation in intestinal crypts and exosomes have been reported to regulate apoptosis.

In their study, Wang et al. [54] compared the peptidomic structure of exosomes de-rived from term versus preterm milk and their effects on IEC and NEC animal models. [54]. Preterm milk improves intestinal epithelium cell migration and proliferation, with higher exosome levels observed. Human normal IEC lines (FHCs) absorb both term and preterm milk exosomes, thereby increasing cell proliferation and migration. The protein expression of term and preterm human breast milk exosomes are different; still, lactotransferrins released from epithelial cells stimulate fibroblast migration, suppress apoptosis, and may regenerate a damaged intestinal mucosa [54,129].

Goblet cells function in the mucosal formation process, significantly contributing to the function of the intestinal barrier [91]. The intestinal barrier that borders the surface of ICEs includes the mucosal barrier, comprising mucus released by goblet cells to aid in the defense against microbial invasion and potentially harmful substances [207]. The mucosal layer lining the intestines, including the mucin secreted by goblet cells, prevents harmful antigens or bacteria from sticking to the intestinal epithelium. Researchers discovered that the oral delivery of human breast milk exosomes increased NEC-protective mucin 2 (MUC2), REGIIIγ, MYD88, and GATA4 expression, whereas the intestines of patients with NEC showed lower mucin levels [208,209]. Both human milk and pasteurized donor milk in preterm infants through exosome content preserved goblet cells and mucosal production that were impaired in NEC [91].

The key connecting factor between ICEs, TJs, is essential for preserving epithelial cell polarity and controlling intestinal barrier permeability [210]. In a study, both the milk of mothers with preterm and term infants restored protective intestinal epithelial TJ proteins (ZO-1, claudin-1, and occludin) in the presence of NEC, showing positive effects in NEC prevention by decreasing inflammation and damage to the intestinal epithelium [129].

Stress exposure in mother mice has been shown to reduce miRNA-148a expression, which in turn has been linked to reduced intestinal barrier function in infants breastfed by stressed mothers, and lowered ZO-1 levels in the ileum of neonatal mice [211].

In vitro and in vivo studies have examined the effects of human breast milk exosomes on ICs and reported that human breast milk exosomes collected at different periods have positive effects; however, the most effective one is colostrum [128]. The effects of raw and pasteurized human breast milk exosomes are similar [91]. Evaluating term and preterm human breast milk exosomes revealed no difference in total protein expression [129], and different proteins were expressed according to a study by Wang et al. [54] (Table 3).

In in vivo and in vitro studies conducted with various animal milk exosomes to ex-amine the effects of exosomes on the intestine, the following results were observed: rat milk exosomes supported IEC viability, increased proliferation, and stimulated ISC productivity [17]; bovine milk exosomes (especially colostrum) significantly reduced caspase 3 activity, an indicator of apoptosis [197]; and yak milk exosomes further activated the PI3K/AKT/C3 signaling pathway, which decreased intestinal inflammation frequency and intensity [198] (Table 3).

Studies on human breast milk exosomes showed that exosomes are effective against LPS-induced epithelial damage in intestinal organoids, and colostrum showed the best protective effect [128], promoted IEC proliferation and migration [54], protected ISCs from the impact of oxidative stress in vitro (a possible pathway of Wnt/b-catenin signaling) [52], decreased intestinal inflammation and increased IC proliferation [199], more markedly restored the number of ileal crypts in vivo, and inhibited inflammatory response in vitro [205,206] (Table 3).

**Figure 5:**
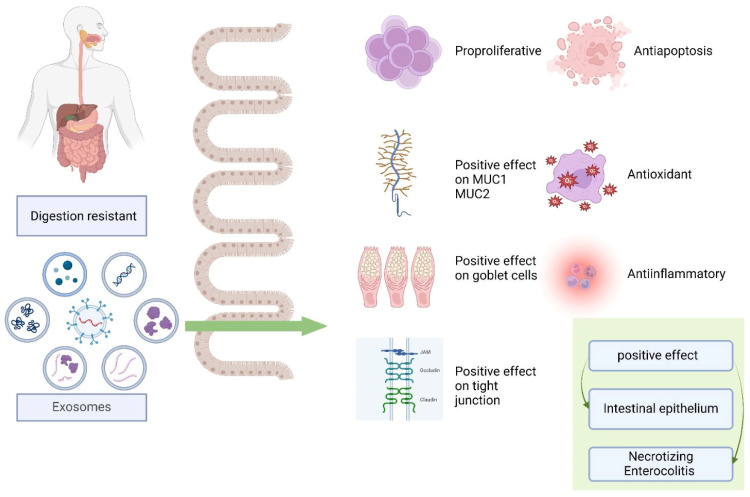
Effects of exosomes on the intestinal epithelium and necrotizing enterocolitis. Abbreviations: MUC1: mucin 1, MUC2: mucin 2.

The study of Shang et al. [212] aimed to locate and describe miRNAs in exosomes from mammals. Using Illumina sequencing technology, miRNAs in the exosomes of donkey, bovine, and mammalian human milk were sequenced. New miRNAs, including 256 in human milk, 346 in bovine milk, and 196 in donkey milk, were identified in human, bovine, and donkey milk exosomes, all tagged with target genes [212]. Mammals share several milk miRNAs but exhibit taxon-specific miRNA fingerprints [100]. Milk-derived miRNA-148a-3p (miRNA-148a) has been linked to the development of newborns’ intestines and is prevalent in human milk [211]. Martin et al. [40] observed that miRNA125b suppressed apoptosis inducer P53, and miRNA148b was associated with IEC growth and survival [40]. Guo et al. reported that that miRNA miR-148a-3p too showed its protective effect against NEC by regulating p53 and SIRT1 [130]. Considering the current literature regarding the possible NEC anti-inflammatory, anti-apoptotic, pro-proliferative, and regenerative effects on ICs are mostly mediated by miRNAs (Figure 5). Wang et al. [54] reported that, unlike miRNAs, preterm milk exosomes increased IEC proliferation and migration due to peptides [54]. miRNA125b suppressed apoptosis inducer p53, and miRNA148b was associated with IEC growth and survival [40]. Another study assessed the impact of human-milk-derived exosomes on mice with NEC, and identified the miRNAs and mechanisms in human breast milk exosomes. The protective effects of miRNA-148a-3p, the most enriched miRNA in human breast milk exosomes, on NEC by regulating p53 and SIRT1 were investigated; it was reported that the in vivo use of miRNA-148a-3p agomir had a mitigating effect similar to human breast milk exosomes and also caused epithelial mesenchymal transition and cell proliferation in normal cells [124,130] (Table 3).

## 5. Future Directions of Human Breast Milk Exosomes in Dietetics

The science of exosome metabolism and biology has become a rapidly expanding and developing field that is open to new discoveries. Along with the discovery and significance of exosomes, their possible effects on diseases are being evaluated, and a break-through is expected in the near future.

For years, human breast milk has biological significance for infants. Human breast milk contains macronutrients, including lactose, oligosaccharides, proteins, and fats. Additionally, it contains minerals, including calcium, magnesium, iron, phosphorus, and zinc, as well as vitamins D and K. In addition to growth factors, macro-, and micronutrients, hormones such as IGF-1, IGF-2, and adiponectin are other components noted in hu-man breast milk, and there is accumulated literature focusing on these nutritional components of human breast milk. Recently, exosomes have been packaged in human milk, and these exosomes are vesicle-style packages containing noncoding RNAs, tetraspanins, oligosaccharides, and other peptides and proteins. Components detected in exosomes can be transferred to infants and cause epigenetic changes. Consequently, they affect the control of cellular processes and gene transcription in various tissues. Therefore, exosomes are associated with several pathways, including the immune system, oxidative stress, and cell cycle.

Several studies have explained the mechanism of how exosomes are produced [35,37,38]. The time-related migration profile and biological functions of human breast milk exosomes in the body are warranted to be mapped. It is of utmost importance to re-veal how orally ingested human breast milk exosomes are distributed and absorbed in the intestine to possess information about the biological effects of their contents.

Exosomes appear in different quantities and varieties at different stages of lactation and time of birth. Examining the effects of miRNAs expressed according to these stages on the pathways more specifically is significant. Moreover, integrating exosomes, of which the effects on pathways have been described, into the clinical field for use in diagnosis and treatment is an issue that should be emphasized in the future. Additionally, the effects of each component in the exosome are separately evaluated in various studies. Under physiological conditions, exosomes are whole. Therefore, when examining the effect of a component, paying attention to its interactions with other components in this process is necessary. For example, when examining the effects of miRNAs in exosomes, they should actually be considered as the exosome matrix. Evaluating one component in isolation may limit the generalizability of the results. cirRNAs detected in human breast milk exosomes can change their expression by binding to miRNA. It is just like a deficiency in evaluating a nutrient in a food on its own and ignoring its interactions with other nutrients.

The potential for drug delivery, physiological activity, safety, and biocompatibility of milk exosomes makes them highly suitable for various cancer treatment applications. To show how certain bioactive exosome components perform biological functions and elucidate any potential side effects from exosome therapy in anticancer activity, future research is needed.

Considering that studies have not yet reached the clinical level, the pipeline should continue for human breast milk exosomes to become an alternative treatment for gut health and NEC occurrence or prevention. Human-milk-derived exosomes could be-come an alternative treatment for NEC prevention in newborns who were not breastfed. Human-milk-derived exosomes have exceptional bioactivity, making them a viable therapeutic option for NEC. The clinical investigation of the role of exosomes, a readily available and natural supply, in cell protection may increase alternative treatment options. The concentration and timing of exosomes will be significant points in understanding this process.

Human breast milk exosome extraction and isolation is a difficult process. The incubation, freezing, and thawing of exosome components, including miRNAs in the laboratory environment and enzyme application, may be among the difficulties present. However, highly sensitive and selective measurement techniques will be significant in detecting exosome components. Optimizing and standardizing techniques for isolation methods and addition to nutritional products such as infant foods and formulas will be essential. Furthermore, performing the metagenomic, metatranscriptomic, and metabolomic profiling of human breast milk, as well as evaluating and profiling human milk viromes and fungomes that may affect human breast milk exosomes will be essential.

Adding miRNAs isolated and extracted from human breast milk to infant foods and formulas will be the subject of future studies. At this stage, in addition to miRNAs, adding bioactive components such as cDNAs, siRNAs, proteins, and plasmid DNAs (antioxidant enzymes) will also be significant. Along with adding them to infant foods, these bioactive components can also be supplemented in infant formulas for infants with miRNA deficiency who receive insufficient or no human breast milk, as well as in the treatment of infants with miRNA-associated diseases; this can be investigated as a method to support pharmacological treatment.

Furthermore, lyophilization could be a proper solution to avert exosome and especially beneficial miRNA degradation caused by the present reservation procedures. Lyophilized human breast milk supplementation to increase the quality of human breast milk substitutes is warranted.

## 6. Conclusions

Human breast milk is an excellent food for babies because it contains various beneficial bioactive components. Each component in human breast milk positively affects human health and has an impact on health through various metabolic interactions. Exosomes are one of the important components of human breast milk. Exosomes can play an important role in intercellular communication and the regulation of homeostasis. Exosomes are vesicle-style packages containing noncoding RNAs, miRNAs, tetraspanins, oligosaccharides, and other peptides and proteins. In addition to exosomes, most studies have focused on the miRNA content of milk exosomes. The exosome and miRNA profiles of individuals are affected by many factors such as stress, obesity, chronic diseases such as diabetes, preterm birth, and type of delivery. Through the components contained in exosomes, epigenetic changes may affect cellular processes in various tissues, control of gene transcription and cellular processes. In relation to this, exosomes can be associated with various pathways, including the immune system, inflammation, oxidative stress, apoptosis, and the cell cycle. Specifically, through immunological regulation, the miRNAs in human breast milk have emerged as viable immune-regulatory agents that target immune cells and affect how an infant’s immune system develops. Moreover, by specifically targeting DNA methyltransferases, miRNAs are essential for regulating the expression of a large number of genes. Identifying the target sites of exosomes and exosome-derived miRNAs in breast milk will help elucidate the effects of these components on the mechanism of many diseases such as obesity, diabetes, NEC, cancer, the immune system disorders, etc. In this review, the factors affecting exosomes obtained from human breast milk, the possible mechanisms of exosomes in various diseases, and the roles of exosomes in infants’ health and dietetics were included. Future research is needed to show how specific bioactive components found in human breast milk exosomes perform biological functions, and to elucidate any uncertain or potential side effects that may arise from exosome treatment. It would also be useful to establish a standardized method for isolating exosomes from breast milk for both clinical and industrial studies.

## Figures and Tables

**Table 1:** Maternal-associated factors affecting human breast milk exosomes.

Maternal-Associated Factor	Mechanism of Action	Reference
Mode of birth		
Cesarean section	Change in miRNA expression levels	[105]
	Greater expression of miRNA-320 than miRNA-148a in colostrum from mothers not receiving exogenous oxytocin	[102]
	Lower levels of miRNA-148a and miRNA-125b in transition and mature human breast milk	[106]
Vaginal birth	Increased in miRNA-148a and miRNA-30 levels, and decreased miRNA-320 levels in colostrum due to increased levels of exogenous oxytocin	[105]
Maternal nutrition		
High-fat or high-carbohydrate diets with similar energy and protein	Increased expression of miRNA-67 and miRNA-27	[107]
	Association with miRNA-148a-5p and miRNA-146b-5p levels related with maternal weight	[83,86,109]
Animals fed the obesogenic diet model	Increased levels of miRNA-222, and decreased levels of miRNA-200 and miRNA-26	[111]
Maternal stress (psychological distress)	Interaction with epigenetic regulation of pathways such as fatty acid metabolism, steroid biosynthesis, and the Hippo signaling pathway	[113]
Maternal weight		
Overweight/obesity	Decreased levels of miRNA-148a and miRNA-30b which are linked to infant anthropometric measurements in the breast milk of overweight/obese women	[115]
Normal body weight	Decreased levels of leptin, adiponectin, and partially different miRNA levels (miR-17, miR-103, miR-181a, miR-let7c, miR-222, miR-146b) in mothers with normal body weight	[109]
Obesity	Changes in 19 miRNAs, including miRNA-575, miRNA-630, miRNA-642a-3p, and miRNA-652-5p, related with neurological diseases and psychological disorders in the breast milk of obese women	[114]
Maternal chronic stress		
Gestational diabetes mellitus	Decreased levels of miRNA-148a, miRNA-30b, miRNA-let-7a, and miRNA-let-7d levels in mothers with GDM, correlated positively with maternal obesity	[112,115]
T1DM	Altered levels of several miRNAs related with proinflammatory cytokine production in the breast milk of mothers with T1DM	[101]

Abbreviations: miRNA: micro ribonucleic acid, GDM: gestational diabetes mellitus, T1DM: type 1 diabetes mellitus.

**Table 2:** Studies examining the possible effects of milk exosomes on cancer.

Type of Milk and Exosome	Cancer Type	Possible Mechanism	Reference
Human breast milk exosome TGF β2	MCF7 breast cancer cells and MCF10A fibrocystic normal cells (derived from woman with benign breast disease)	TGF β2 dose-dependent (high dose); Affects cell morphology and actin cytoskeleton Increases SMA and vimentin levels and decreases E-cadherin levels Promotes EMT	[55]
Human breast milk exosomes	LS123 colon cancer cells	Milk-derived exosomes can penetrate both malignant and normal cells, and change the mRNA expression profiles of those cells Following incubation, the miRNA-148a expression was upregulated, decreasing tumor cell proliferation	[124]
Camel milk exosomes	MCF7 breast cancer cells	Inhibited MCF7 cell proliferation Tumor weight decreased Tumor DNA damage increased Higher caspase-3 activity, increased Bax, and downregulated Bcl2 gene expression all pointed to cancer cell death via apoptosis Inhibition of malondialdehyde levels and inducible nitric oxide synthase mRNA levels; higher levels of catalase, superoxide dismutase, and glutathione peroxidase in tumor tissues Decreased NF-κB, IL-1β, MMP9, VEGF, and ICAM1 gene expression levels Reduced metastasis and angiogenesis in tumor tissues	[148]
Buffalo milk exosomal miRNA-27b	HCT116 and HT-29 colorectal cancer cell	miRNA-27b transfection increased cytotoxic effects Lysosome accumulation and the amount of reactive oxygen species in the mitochondria were increased in miRNA-27b^+^ cells miRNA-27b promoted mitochondrial stress and apoptotic death	[149]
Bovine milk lactoferrin-loaded exosomes (exoLF)	MDA-MB-231 breast cancer cell line	Cytotoxic on cancer cell while normal mesenchymal stem cells remained viable A decrease in anti-apoptotic protein Bcl-2 levels and an increase in the pro-apoptotic protein Bid levels following the exoLF therapy	[150]
Bovine milk exosomes	A549 and H1299 lung cancer HCT116 colon cancer PC3 and DU145 prostate cancer MDA-MB-231 and MCF7 breast cancer PANC1 and Mia PaCa2 pancreatic cancer OVCA432 ovarian cancer	Inhibits the proliferation of certain types of human cancer cells (antiproliferative effect)	[154]
Anthocyanidin-loaded exosomes (ExoAnthos)	A549 and H1299 lung cancer HCT116 colon cancer PC3 and DU145 prostate cancer MDA-MB-231 and MCF7 breast cancer PANC1 and Mia PaCa2 pancreatic cancer OVCA432 ovarian cancer	Antiproliferative effects ExoAnthos exhibited a dose-dependent inhibition of TNFα-induced and NF-κB activity in breast (MCF7) and lung (H1299) cancer cells	[154]

Abbreviations: EMT: epithelial–mesenchymal transition, SMA: smooth muscle actin, TGF β2: transforming growth factor-beta2, miRNA: micro ribonucleic acid, mRNA: messenger ribonucleic acid, DNA: deoxyribonucleic acid, NF-κB: nuclear factor-κappa B, IL-1β: interleukin 1beta, MMP9: matrix metalloproteinase, VEGF: vascular endothelial growth factor, ICAM: intercellular adhesion molecule 1, exoLF: lactoferrin-loaded exosomes, TNFα: tumor necrosis factor-alpha, ExoAnthos: anthocyanidin-loaded exosomes.

**Table 3:** Studies examining the effects of milk exosomes on epithelial damage and necrotizing enterocolitis.

Type of Exosome	Cell Culture or Experimental Animal	Possible Mechanism	Reference
Resistance to digestion			
Human milk miRNA 22-3-p	In vitro human intestinal crypt-like cells (HIEC)	Exosomes remained viable in in vitro digestion and absorbed by ICs. Overall, 288 mature miRNAs were isolated from samples of exosomes in the intestinal epithelium. Hsa-miRNA-22-3p was the most prevalent miRNA. Both digested and undigested exosomes entered the cells of the human intestinal crypt.	[53]
miRNA bovine milk formula	In vitro HIEC-6 In vivo newborn piglets	Milk-derived miRNAs survive gastrointestinal passage in newborns, accumulating in preterm piglets’ ICs after enteral feeding with bovine colostrum/formula. In piglets, supplementing colostrum with cel-miR-39-5p/-3p raised blood concentrations of cel-miR-39-3p and AGO2 loading in ICs. This shows that miRNA signaling could be transmitted vertically from milk to the newborn digestive tract.	[100]
Sahiwal cow-derived exosomal miRNA	In vitro Caco-2 cell	Exosomes isolated from in vitro digested milk and their respective water controls (miRNA-182-5p, miRNA-148a, miRNA-25, miRNA-21, and miRNA-2478) were among the immune-related miRNAs that did not significantly differ. Milk exosomal miRNAs can cross the intestinal barrier meaning these are resistant to digestion. Transepithelial migration via the Caco-2 monolayer.	[193]
Holstein cow-derived exosomal protein Colostrum Milk	In vitro Caco-2 cell	The study examined the expression of exosome surface indicators in the colostrum and milk of H, A, and L immune responder cows, revealing their ability to be absorbed by human intestinal epithelial cells. Co-incubation with colostrum and milk exosomes from H responder cows resulted in significantly higher metabolic activity compared to L responder exosomes. Milk exosomes, unlike colostrum exosomes from L responders, did not activate the caspase 3 pathway in Caco-2 cells, as evidenced by significantly lower caspase 3 activity, an indicator of apoptosis.	[197]
Protective effects on epithelial function			
Human breast milk exosomes	In vitro ISCs	The vitality of ISCs was significantly improved by adding exosomes to ISCs exposed to H_2_O_2_. Marked upregulation of the Wnt/b-catenin axis genes Cyclin D1, c-Myc, and Axin2 mRNA expression in ISCs treated with exosomes (*p* < 0.05 for all). The inclusion of carnosic acid, a specific Wnt/b-catenin signaling inhibitor, markedly decreased the viability of the cells. ISC was protected from oxidative stress damage.	[52]
Human breast milk exosomes Colostrum Transition milk Ripe milk	In vivo C57BL/6 mice pups Ex vivo intestinal organoid model	Intestinal organoids shrank in size when exposed to LPS, which also promoted intestinal regeneration and inflammation by TNF-α and TLR4 expression upregulation. Human-milk-derived exosomes protected the intestinal organoids from LPS-induced epithelial damage. Colostrum was more effective protective agent, suppressed LPS-induced injury and reduced inflammation (TNF-α and TLR4 expression reduction) than other term milks.	[128]
Holstein cow exosomal proteins Colostrum Milk	In vitro Caco-2 cell culture	Exosomes from cow colostrum and milk preserved Caco-2 metabolic function and did not harm these cells. Colostrum suppressed the activity of the apoptosis marker caspase 3.	[197]
Exosomal proteins yak vs. cow milk	In vitro IEC-6	The proteomics examination found 58 higher and 334 lower expressed proteins in yak milk exosomes compared to cow milk exosomes. Compared with cow milk exosomes, yak milk exosomes more efficiently activate the PI3K/AKT/C3 signaling pathway, thereby increasing IEC-6 survival and decreasing intestinal inflammation incidence and severity. Milk exosomes achieve this effect in two ways, which are attenuated LPS-induced intestinal inflammation and decreased inflammatory cytokine levels	[198]
Human breast milk exosomes	In vivo Sprague–Dawley rats	Exosomes administration decreased the damage caused by intestinal IR. In rats with IR, exosome injection resulted in a considerable TNF-α downregulation. Exosomes promoted intestinal regeneration, which decreased IR-mediated damage. Ki67 protein expression was significantly elevated with exosomes, suggesting enhanced IEC proliferation. Exosomes mitigated IR-induced intestinal damage by boosting intestinal regeneration and lowering mucosal inflammation.	[199]
Possible effects on NEC			
Human breast milk exosomes miRNA125b miRNA148B	In vitro IECs	miRNA125b suppressed p53, which is an apoptosis-inducing agent. miRNA148b was associated to IEC development and survival. Exosomes prevented IECs from destruction. Exosomes supported gastrointestinal and immune system development.	[40]
Human breast milk exosomes miRNA 22-3-p	In vitro HIEC	In the intestinal epithelium, 288 mature miRNAs from all 24 exosome samples were detected. Exosomes provided oxidative stress resistance for human intestinal crypt-like cells.	[53]
Human breast milk exosomes Exosomal peptides Preterm milk Term milk	In vitro FHC In vivo Sprague–Dawley rats	Compared with term exosome application, preterm exosome application significantly increased FHC proliferation and cell migration. In the in vivo study, three proteins were upregulated about the topic. Two of them with immunomodulatory, anti-inflammatory and antimicrobial properties were lactoferrin and one of them with potentially regenerating the damaged intestinal mucosa properties was one lactoadherin MFGE8.	[54]
Human breast milk exosomes Raw milk Pasteurized milk	In vitro Ex vivo C57BL/6 mice	Administration of exosomes from raw and pasteurized human breast milk during NEC decreased IL-6 expression and MPO activity, and increased goblet cell count. MUC2 expression was elevated by both milk, with no discernible difference in MUC2 expression between the two. NEC-induced inflammatory response was equally attenuated by raw and pasteurized human breast milk exosomes. Protected goblet cells and mucosa production.	[91]
Human breast milk exosomes miRNA-148a-3p	In vitro Normal colonic epithelial cells Colonic tumor cells	Exosomes in normal cells downregulated PTEN, a miRNA-148a target, inhibiting proliferation and DNMT1 expression, inducing alterations associated with epithelial mesenchymal transition. Exosomes did not exhibit this effect on tumour cells.	[124]
Human breast milk exosomes Preterm milk Term milk	In vitro Caco-2 cell line In vivo C57BL/6 mice	LPS damage severely damaged the intestinal mucosa in pups, whereas human-milk-derived exosomes offered protection. NEC and NEC + milk without exosomes showed higher proinflammatory cytokine IL-6β and TNF-α levels. NEC + milk without exosomes showed lower ZO-1, claudin-1, and occludin levels.	[129]
Human breast milk exosomes miRNA-148a-3p	In vitro Small IEC line (IEC-6) In vivo C57BL/6 mice	By reducing target p53 expression, miRNA-148a-3p increased SIRT1 levels. The in vivo use of miRNA-148a-3p agomir showed a similar protective effect as human breast milk exosomes.	[130]
Human breast milk exosomes AFSC exosomes	In vitro IEC-6 rat crypt epithelial cells IEC-8 rat ileal epithelial cells In vivo C57BL/6 mice pups	Human AFSC exosomes vs. Human breast milk exosomes (in vitro): AFSC exosomes selectively controlled IEC migration, whereas human breast milk exosomes preferentially suppressed the inflammatory response of IECs. Human breast milk exosomes (in vivo): The number of ileal crypts was markedly recovered in vivo following human milk exosome administration compared with that using AFSC exosomes.	[205]
Human breast milk exosomes	In vitro IEC-6 rat small intestinal epithelial cells In vivo Sprague–Dawley rats	Intravenous or enteral exosome therapy significantly decreased the frequency and intensity of experimental NEC (29% vs. 11.9%) (in vivo), while safeguarding IECs from damage by anti-apoptopic and pro-proliferative properties (in vitro).	[206]
Human breast milk exosomes mir-148a	In vitro Caco-2 cell In vivo CD1 mice	Caco-2 cells showed that ZO-1 protein levels were markedly increased, whereas DNMT1 protein levels were significantly decreased upon miRNA-148a overexpression (in vitro). With an upward trend in DNMT1 levels in infant intestines, stress-induced suppression of miRNA-148a expression in mothers’ milk may result in a decrease in intestinal ZO-1 concentration (in vivo).	[211]

Abbreviations: ICs: intestinal cells, HIEC: human intestinal crypt-like cells, AGO2: argonaute RISC (RNA-inducing silencing complex) catalytic component 2, miRNA: micro ribonucleic acid, ISCs: intestinal stem cells, H_2_O_2_: hydrogen peroxide, LPS: lipopolysaccharide, TNF-α: tumor necrosis factor-alpha, TLR4: Toll-like receptor 4, IEC: intestinal epithelial cell, IR: ischemia–reperfusion, FHC: human normal IEC lines, MFGE8: milk fat globule EFD factor 8/lactadherin, SIRT1: sirtuin 1, NEC: necrotizing enterocolitis, MUC2: mucin 2, MPO: myeloperoxidase, PTEN: phosphatase and tensin homolog, IL-6β: interleukin 6beta, AFSC: amniotic fluid stem cell, ZO-1: zonula occludens-1, DNMT1: DNA methyltransferase 1.

## Data Availability

Not applicable.
